# French guidelines for the management of nonadvanced mastocytosis in adults

**DOI:** 10.1186/s13023-025-03764-7

**Published:** 2025-10-02

**Authors:** Cristina Bulai Livideanu, Stéphane Barete, Ghandi Damaj, Michel Arock, Julien Rossignol, Olivier Hermine

**Affiliations:** 1 Department of Dermatology, CEREMAST Toulouse, Toulouse University Hospital, Paris, France; 2 CEREMAST Pitié-Salpêtrière, AP-HP, Paris, France; 3 Department of Hematology, Caen University Hospital, Caen, France; 4 Department of Hematology, CEREMAST Necker, AP-HP, Paris, France

**Keywords:** Mastocytosis, Therapy, NPCD, Recommendations, Expert center

## Abstract

Mastocytosis is a rare disease caused by abnormal mast cell accumulation/proliferation in various organs/tissues. Its clinical features exhibit significant variability irrespective of its clinical presentation, classification, global survival or impact on quality of life (QOL). For example, 15% of adult patients present isolated skin mastocytosis, called cutaneous mastocytosis (CM), while 85% have systemic mastocytosis (SM), with the most frequent type of skin lesions being systemic mastocytosis with cutaneous infiltration. In addition, regardless of the location of the mast cells or whether the CM or SM is isolated (whether with or without associated skin involvement), symptoms due to mast cell activation can vary in severity and may be life-threatening. All these manifestations can impose additional stress on patients.

This protocol is a compendium of evidence-based recommendations formulated by experts. Its purpose is to assist clinicians in the management of these rare and frequently complex diseases. It describes the management of adult patients with nonadvanced or indolent mastocytosis (CM), CM with primary MCAS, indolent systemic mastocytosis, bone marrow mastocytosis, and smoldering mastocytosis.

**Supplementary Information:**

The online version contains supplementary material available at 10.1186/s13023-025-03764-7.

## Aims

The objective of this care and diagnosis national protocol (CDNP) is to present to health professionals the current optimal diagnostic, management and care recommendations for adult patients presenting with indolent mastocytosis, whether cutaneous mastocytosis (CM), CM with primary MCAS, indolent systemic mastocytosis (SM), bone marrow mastocytosis (BMM), or smoldering SM (SSM).

The aim of the CDNP is to standardize the treatment and monitoring of the disease to improve the quality of life (QoL) of patients and those around them.

The CDNP cannot, however, consider all specific cases, all comorbidities, all therapeutic particularities, hospital care protocols, etc. It cannot claim to be exhaustive for possible treatment methods or replace the individual responsibility of doctors toward their patients. However, this protocol reflects the essential approach to managing patients with mastocytosis and will be updated as new data are validated.

## Method

This protocol has been developed according to the recommendations published in June 2022 by the French National Authority for Health, on the basis of a literature review, with the help and validation of members of the French Network for Rare Immuno-hematological Diseases (MaRIH) (Table S1).

### Mastocytosis: general background

Mastocytoses constitute a heterogeneous group of diseases with abnormal accumulation/proliferation and activation of pathological and clonal mast cells (over 90% of cases) in one or more organs or tissues (skin, bone marrow, gastrointestinal tract, bones, and lymph nodes) [1]. These are rare diseases that were initially included among the myeloproliferative syndromes. More recently, mastocytosis has been differentiated from the latter [2–4]. Nonadvanced mastocytosis is in fact more likely to be a chronic inflammatory disease than a myeloproliferative syndrome. The clonality of mastocytosis is linked to an acquired “gain-of-function” mutation in the gene encoding the KIT receptor, a transmembrane receptor having tyrosine kinase activity.

Mastocytosis has very diverse presentations and variable prognoses, ranging from good to severe. We distinguish between nonadvanced mastocytosis (87%) and advanced mastocytosis (13%), which have opposite prognoses, with almost normal survival but sometimes significant handicap for nonadvanced forms, and reduced survival for advanced forms [5–7]. Mastocytosis affects both children and adults, but its course is often different in the two poulations. In children, mastocytosis is generally limited to the skin and is very rarely systemic. It can begin very early in life but regresses by adolescence in at least 60% of cases [8].

In adults, the disease is most often systemic, although cutaneous forms may be observed (CM) [9]. Mastocytosis manifests in two distinct forms: the first form is characterized by the presence of skin lesions (81% of all cases). The second form, known as systemic mastocytosis, presents without skin lesions and exclusively affects the bone marrow (19% of cases) [10]. Some forms may be extracutaneous, for example, with only a digestive presentation. Mastocytosis in skin (MIS) in adults may occur in skin alone (CM, 15% of cases), and is most often associated with systemic mastocytosis (SM, 70% of cases) or, more rarely, with “CM with primary MCAS” (15% of cases), where the criteria are insufficient in number to establish a strict diagnosis of SM [9]. CM with primary MCAS was described by Valent et al*.* in 2017 [6]. Patients with primary MCAS were described in 2016 by Pardanani [11]. This condition is characterized by the absence of all the diagnostic criteria for SM, with the presence of only one or two minor criteria, including a clonality criterion without the major criterion. It is noteworthy that this conceptualization of primary MCAS has not yet received formal acknowledgement from the WHO.

**Fig. 1:**
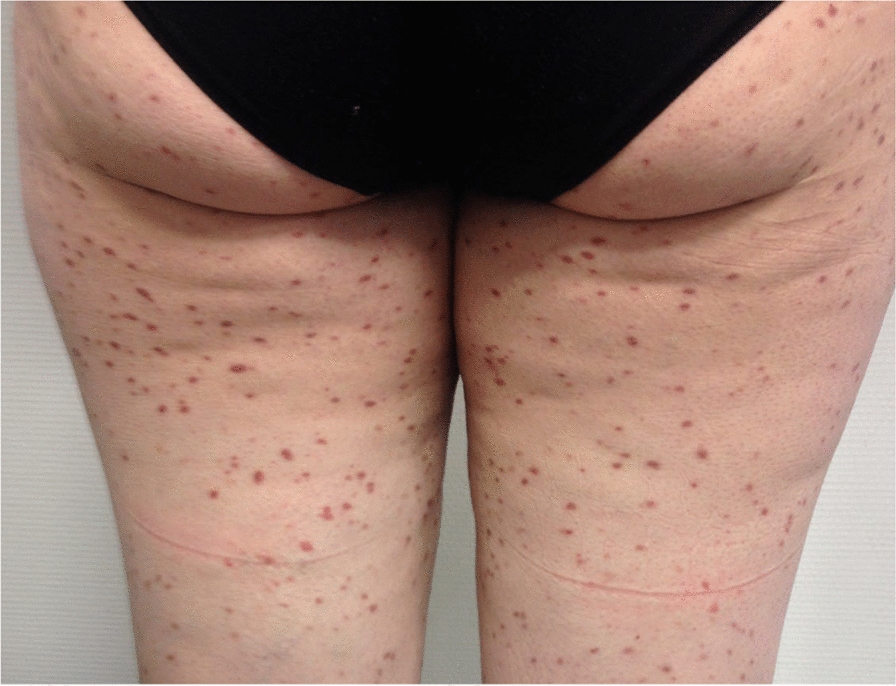
Urticaria pigmentosa cutaneous mastocytosis

**Fig. 2:**
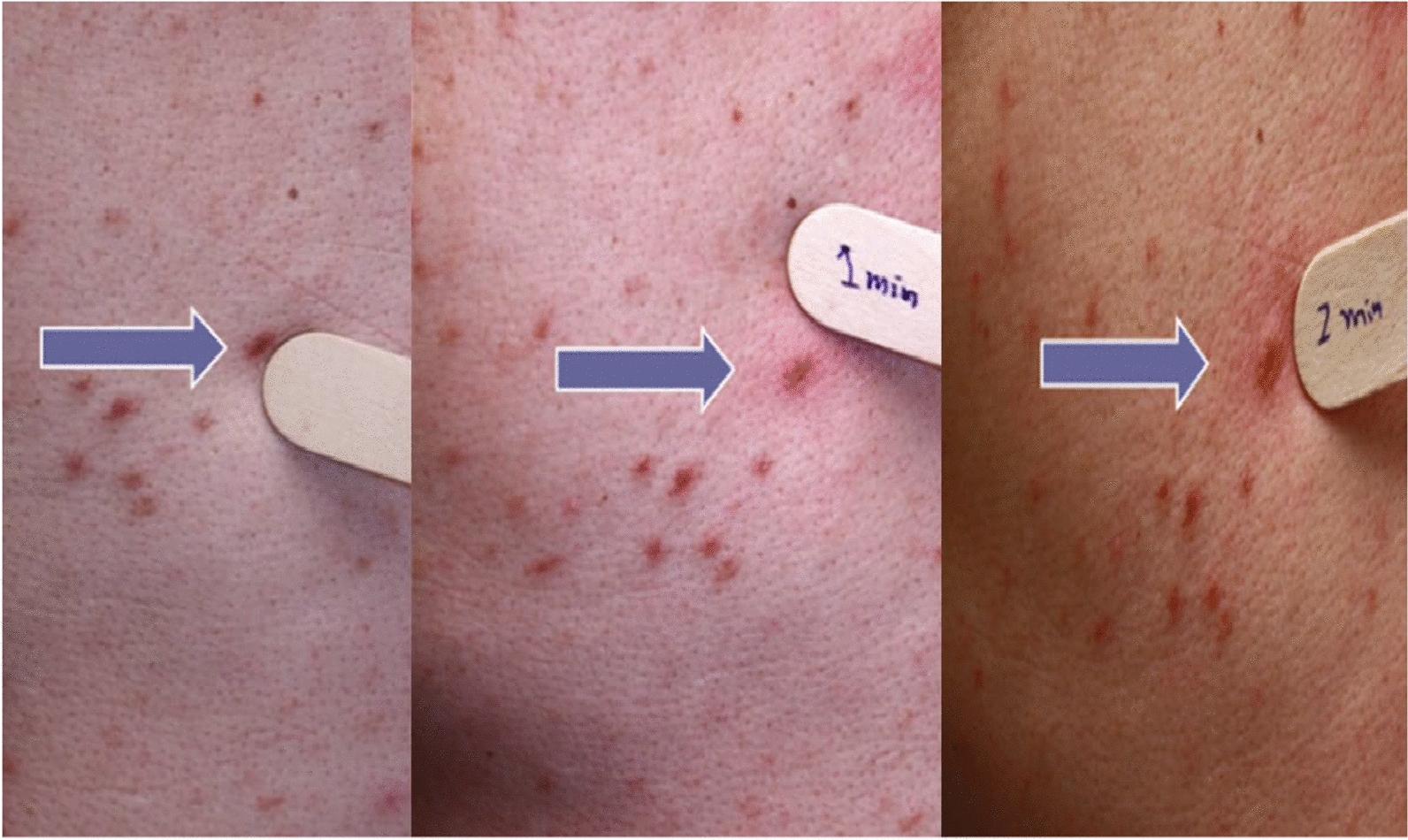
Positive Darier sign in a patient with cutaneous mastocytosis

**Fig. 3:**
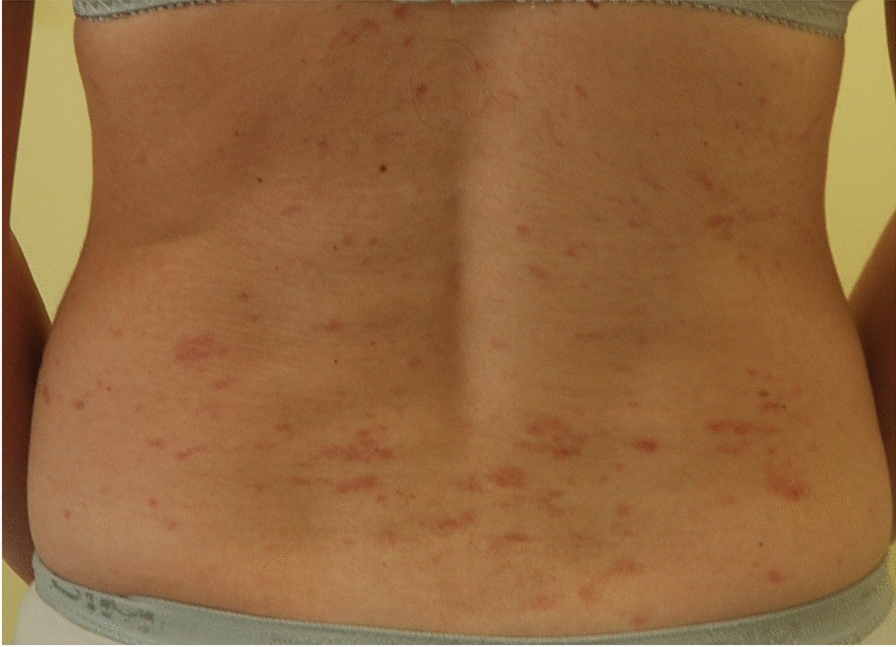
Telangiectatic cutaneous mastocytosis

**Fig. 4:**
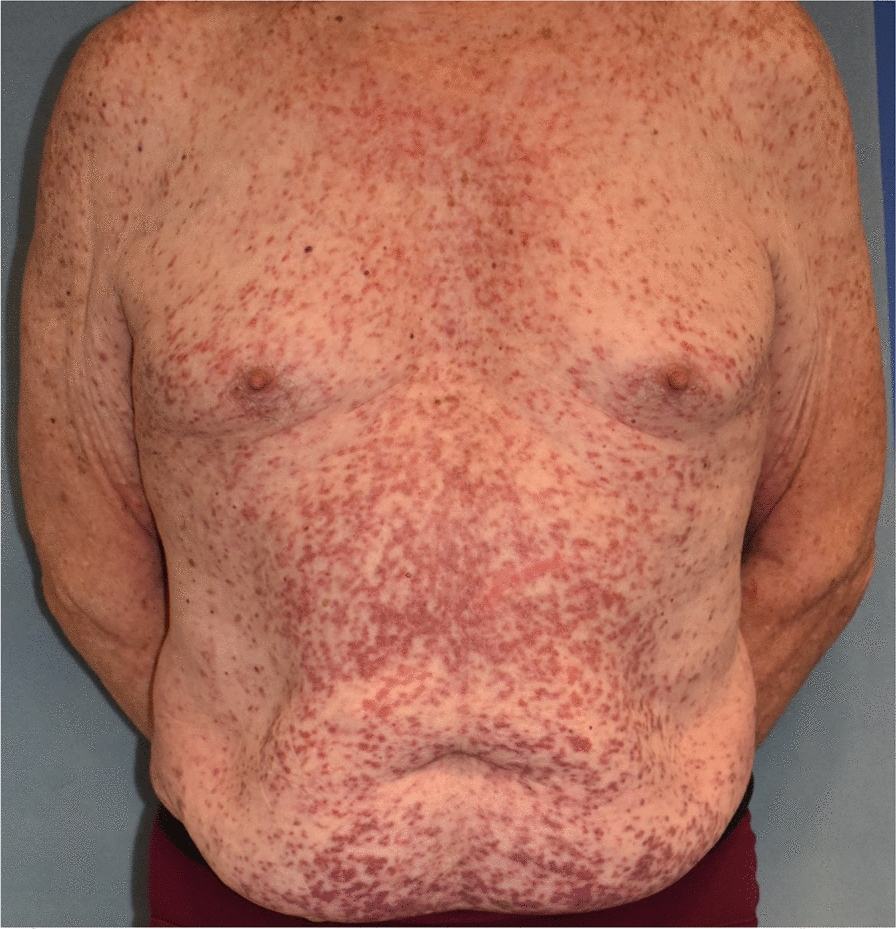
Erythematous cutaneous mastocytosis

The clinical presentations are associated with mast cell activation, the accumulation of mast cells in organs, or both processes concomitantly, and may correspond to the following features:

1. Patients with heterogeneous, recurrent mast cell activation symptoms of variable severity, which may be life-threatening, such as anaphylactic shock;
2. Patients with osteoporosis, with or without pathological bone fractures [12–17];
3. Patients with specific signs linked to the accumulation of pathological mast cells in at least one organ, including cutaneous signs, hepatosplenomegaly/adenomegaly, cytopenias, for example.
4. Patients presenting with the combinations of features 1 + 2 + 3, 1 + 2, 1 + 3 or 2 + 3 given in Table 1 (Figs. 1, 2, 3 and 4).

**Table 1:** Key features of the different types of mastocytosis

The practitioner must consider mastocytosis in adults and refer the patient to a mastocytosis specialist if the following clinical signs appear:
1. Fixed maculopapular pigmented skin lesions of variable extent (Fig. 1), with Darier’s sign, corresponding to a picture of so-called pigmentary urticaria (Fig. 2);
2. Telangiectatic skin lesions on the trunk and upper limbs, with or without Darier’s sign, corresponding to a telangiectatic form called telangiectasia macularis eruptiva perstans (TMEP) (Fig. 3);
3. Erythematous-violaceous skin lesions of varying degrees of diffusion (Fig. 4), with Darier's sign;
4. Presence of symptoms of mast cell activation affecting at least two organs [50] (Table S3), in the absence of any other identified cause and manifesting as flare-ups, often appearing spontaneously and recurrently, and/or chronically;
5. Occurrence of repeated anaphylactic reactions following Hymenoptera stings, in some cases appearing more than one hour after the Hymenoptera sting;
6. Presence of anaphylactic reaction with no discernible etiology (negative allergological investigation);
7. Presence of anaphylactic reaction with a known allergy and a basal serum tryptase level greater than normal and distant from the reaction;
8. Severe anaphylactic reaction to Hymenoptera stings with very low sensitization parameters (very low specific IgE levels, in some cases < 1 kU/L, and/or negative skin tests);
9. Presence of early osteoporosis not accounted for by the initial assessment and/or repeated low-energy vertebral fractures (low trauma);
10. Presence of the above items in the same patient
*What should be done?*
•In the event of a suspicious skin lesion the patient should be referred to a dermatologist for skin biopsy (complete microscopic, immunohistochemical and molecular biology analysis), with systemic assessment if a disability is present (to be assessed)
•Propose first-line symptomatic treatment
•Refer the patient to a mastocytosis competence/reference center for basic treatment
*Involvement of the practitioner in patient monitoring*
•Monitoring of treatment safety and of improvements in symptoms and disabilities
•Monitoring of improvement in quality of life (QoL)

The present CNPD describes the management of nonadvanced mastocytosis cutaneous mastocytosis [CM], CM with primary MCAS, indolent systemic mastocytosis [ISM], bone marrow mastocytosis [BMM], smoldering [slowly progressive] systemic mastocytosis [SSM], and monoclonal MCAS or primary MCAS in adults).

### Pathophysiology of mastocytosis

The pathophysiology of mastocytosis is essentially based on autoactivation of KIT mutations detected in humans. Such mutations induce tyrosine kinase activity in the receptor, even in the absence of the SCF ligand [18]. The genetic study on patient cohorts identified a primary mutation at codon 816 (Asp816Val) in more than 90% of adults, as well as rarer mutations on this same codon 816 (Asp816Tyr, Asp816Phe, Asp816His), but also on other codons [19, 20].

## Clinical presentations of mastocytosis

### Mastocytosis in skin (Table 2)

**Table 2:** Diagnostic criteria for the different forms of cutaneous mastocytosis

Form of CM	UP*	TMEP**	Mixed forms UP and TMEP
Context	Woman or man > 40 years old Pruritus flush, cutaneous discomfort of additive pigmented lesions	Vascular form of UP variant (20%)	Adults
Primary lesions	Brownish maculopapules ± fixed erythematous Distribution on thighs and thorax	Telangiectatic lesions on upper thorax and limbs	Associated Injury
Pruritus	+ + (first symptom)	No pruritus	Yes
Associated signs Darier’s sign	Darier’s sign	Darier’s sign negative	Darier’s sign positive
Diagnosis	Cutaneous biopsy with anti-CD117 (KIT) immunostaining, mast cell count > 20 per window at 40 × magnification Cutaneous biopsy to identify *KIT* D816V mutation in skin Screening for WHO extracutaneous criteria to ascertain presence of cutaneous features of SM or CM with primary MCAS (85%)	Cutaneous biopsy to identify *KIT* D816V mutation in skin Screening for WHO extracutaneous criteria to ascertain presence of cutaneous feature or SM (50%)	Cutaneous biopsy to identify KIT D816V mutation in skin Screening for WHO extracutaneous criteria to ascertain presence of cutaneous features of SM or CM with primary MCAS (85%)
Treatment	Symptomatic and basic treatment in the event of disability or clinical progression patients respond frequently	Symptomatic treatment and vascular treatment for telangiectasia	Symptomatic treatment and vascular treatment for telangiectasia

^*^UP: Urticaria pigmentosa or pigmented maculopapules

^**^TMEP: Telangiectasia macularis eruptiva perstans

#### Clinical signs

Mastocytosis in skin manifests itself as multiple pigmented and inflammatory maculopapular skin lesions of varying extent, which occasionally coalesce into plaques and placards. Darier’s sign is a distinctive diagnostic indicator, albeit not definitive. It manifests as swelling accompanied by an accentuation of the erythema within minutes (3 to 5 min) following the application of pressure from a tongue depressor to one or more pigmented lesions (Fig. 2). The clinical phenotype of these lesions consists of maculo-papules of uniform pigmentation, buff flesh, dark brown and/or red-purplish, and of variable size (monomorphic or polymorphic and of variable size), or erythematous maculo-papules dotted with telangiectasias. The lesions may be localized, for example to skin folds, or they may extend over the entire integument. They rarely affect the face, palmar-plantar regions, genital regions [21] or the scalp. They may be pruritic.

**Fig. 5:**
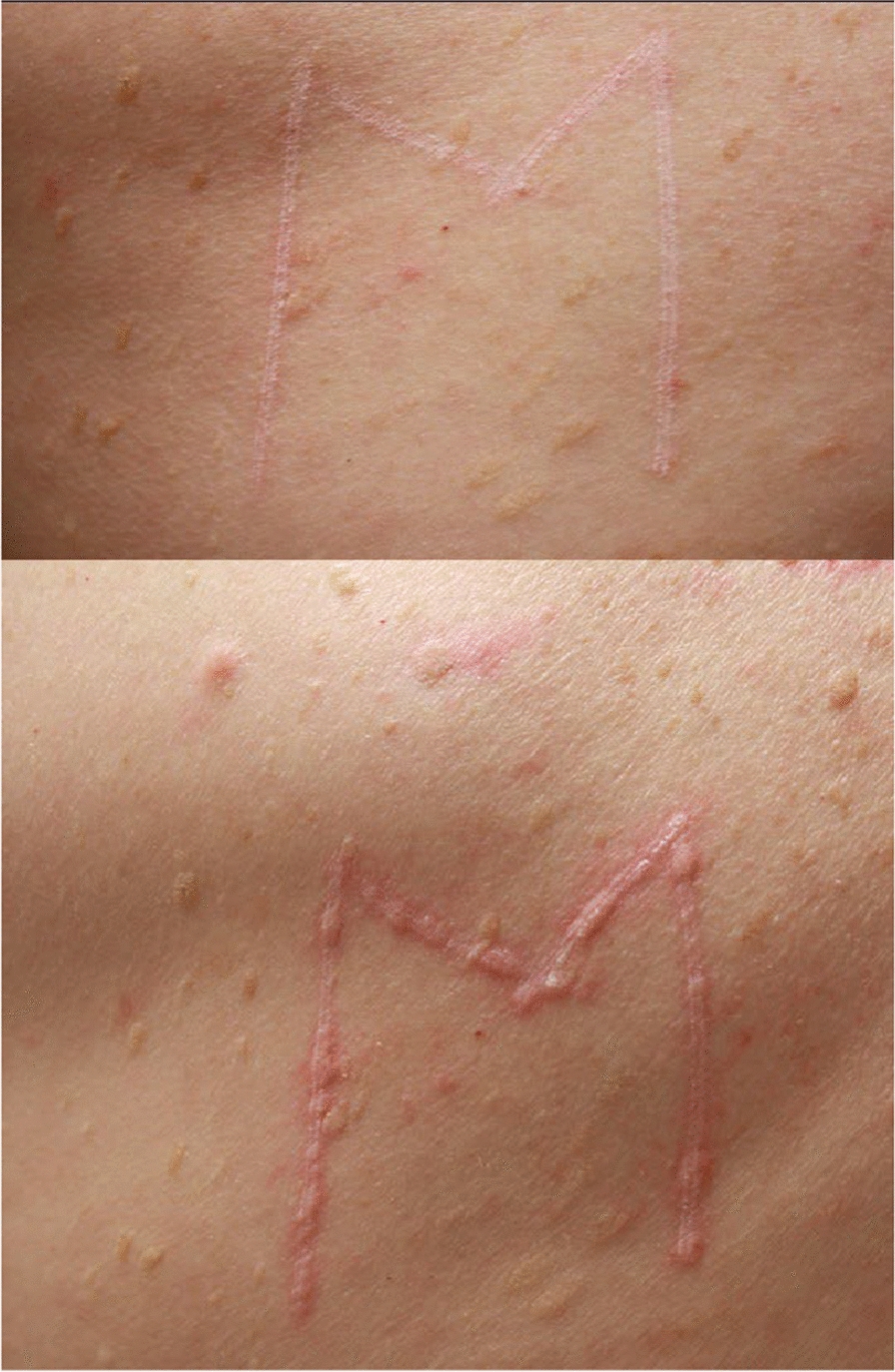
Cutaneous dermography

Nonspecific skin lesions, such as those associated with dermographism, may be present beyond pigmented lesions and these should be sought (20%) [22] (Fig. 5).

### Positive diagnosis of mastocytosis in skin

The diagnosis of mastocytosis in skin is based on two criteria. The primary criterion is the clinical appearance, as outlined in paragraph 2.1.1. Table S2 presents the differential diagnosis of cutaneous mastocytosis. The secondary criterion includes the histological criterion, identified by immunohistochemistry, and the molecular biology criterion, which involves identification of the *KIT* D816V mutation in the skin [23]. Thus, the diagnosis of in skin can be confirmed by a combination of anatomic pathological and immunohistological examination of the skin, with the use of anti-CD117 antibody (anti-KIT) and anti-tryptase Abs, and by identifying the *KIT* mutation in the skin. Biopsies are obtained using 4-mm punches following administration of local anesthesia consisting of simple or adrenalinated xylocaine.

In anatomical pathology, an increase in the number of mast cells in the dermis has been demonstrated [23], with the classic clinical phenotype of more than 20 mast cells per field at high magnification (X 40) [24]. These mast cells may be atypical in appearance, spindle-shaped, and arranged around dilated vessels [25], disseminated in the dermis, and/or grouped in clusters of over 15 mast cells [24]. Mast cells can be more readily identified through the use of immunostaining with anti-tryptase antibody or anti-CD117 antibody [1, 26]. Pigmentation of the epidermal basal layer and the presence of eosinophils in the dermis [27] can facilitate histological diagnosis.

Screening for the *KIT* D816V mutation is best performed using ASO-qPCR or digital PCR (technical consensus in Europe) on DNA extracted from skin lesions via an allele amplification technique specific for the *KIT* D816V mutation [28, 29]. In the event of a negative result, the entire coding sequence of the *KIT* gene can be analyzed on RNA extracted from skin lesions using a sequencing technique (National Center of Reference of Marseille) [30]. Recently, in 43% of urticaria pigmentosa cases, diagnosis was made solely through identification of the *KIT* D816V mutation in the skin because the immunohistochemical examination was negative [31].

As previously stated, MIS in adults can occur in distinct forms: as an isolated CM (15% of cases), in association with primary MCAS (20% of cases), or with systemic involvement (65% of cases) [9].

A novel scoring system has recently been proposed to help assess the risk of developing systemic disease (SM) in patients already diagnosed with mastocytosis in skin [32] (Table S3). This score ranges from  −1 to 5, depending on the serum tryptase (ST) level, the presence of bone pain or osteoporosis and the presence of constitutional or cardiovascular symptoms. A score of  −1 or 0 indicates a low risk of having SM (0.7% to 25%), while a score of 1 or 2 points indicates an intermediate risk of having SM (47% to 71%), and a score of 3 to 5 is associated with a high risk of SM (87% to 98%).

## Systemic mastocytosis

### Clinical signs

In 80% of cases, systemic mastocytosis manifests as skin lesions [10].

Mastocytosis without skin lesions (20% of cases) can present clinically in the following ways:

- Symptoms of mast cell activation (Table S4) involving at least two organs and manifesting recurrently and/or chronically;
- More or less severe anaphylaxis in the absence of identified allergens (idiopathic anaphylaxis);
- Poor tolerance of desensitization to Hymenoptera venom (from bees and wasps), despite a gradual increase in desensitization levels and the exclusion of local reactions;
- Early osteoporosis and/or low-energy vertebral fractures (low trauma) without a classic cause of osteoporosis;
- The presence of cytopenias (anemia, thrombocytopenia or leukopenia) or myelocytosis, or the presence of circulating mast cells, which may lead to a diagnosis of advanced SM;
- Abnormal liver function tests (cholestasis) in the absence of other discernible causes, which may result in a diagnosis of advanced SM;
- Disorders such as malabsorption with hypoalbuminemia and weight loss of more than 10% of the patient’s initial weight in the absence of other causes, resulting in a diagnosis of advanced SM;
- Organomegaly, with hepatomegaly and/or splenomegaly being the most common.

### Positive diagnosis of systemic mastocytosis

SM is confirmed after screening has been carried out (Table S5) [33].

1. Bone marrow biopsy (BMB) is performed to identify the major diagnostic criterion (multifocal dense infiltrate of ≥ 15 aggregated mast cells). BMB may also be used to screen for a minor cytological criterion characterized by the presence of 25% spindle-shaped, degranulated mast cells.
2. Bone marrow puncture for:

- Cytological examination (myelogram) to identify at least 25% of mast cells with abnormal cytology—spindle-shaped, degranulation—among all identifiable mast cells;
- Immunohistochemical examination to identify mast cells (CD117 +) expressing CD25, CD2, and/or CD30 or immunophenotypic examination (flow cytometry) to identify the same minor criterion but with better diagnostic performance (positive predictive values of 96.2% to 100% and negative values ranging from 48.7% to 87.6%) [34];
- Screening for the D816V mutation in the *KIT* gene, which is positive in more than 94% of cases of SM. This research must be carried out by ASO-qPCR or by droplet digital PCR using DNA extracted from blood and bone marrow cells (technical consensus in Europe, quantitative with a detection threshold of 0.01%). These techniques also enable quantification of the tumor mast cell mass (allelic load) by expressing the allelic fraction of the circulating or medullary *KIT* D816V mutation, in particularly to monitor possible cytoreductive treatments (cladribine, TKIs) [28, 29]. If this investigation yields a negative result but the suspicion of systemic mastocytosis remains high, analysis of the entire coding sequence of the *KIT* gene can be performed by sequencing RNA extracted from the bone marrow [30, 35]. The search for the *KIT* D816V mutation can be extended to include other organs, such as the liver, gastrointestinal tract or any other infiltrating internal organ. The utilization of alternative genetic techniques is not advised due to their limited sensitivity.
- Basal serum tryptase measurement in the absence of an anaphylactic reaction; a level > 20 ng/mL is a minor diagnostic criterion for SM. This minor criterion will require re-evaluation in the future given the impact of duplication of the *TPSAB1* alpha-tryptase gene (present in 5% of the general population) on the basal tryptase level [36].
  This latter condition is documented in the WHO criteria [37], but not in the ICC criteria [38]. Table S6 sets out all differences between the diagnostic criteria of WHO 2022 and ICC 2022. Similarly, Table S7 presents the differences in the WHO 2022 and the ICC 2022 classifications of mastocytosis [37, 38].
  Although not yet officially included in the international diagnostic criteria for SM, other criteria, such as tryptase levels in bone marrow determined using the classic technique, are useful for diagnosis [9, 39]. Nevertheless, the diagnostic value of this novel criterion must be substantiated through prospective studies. Although this has been done, the extant data have not yet been published.

### Summary for general practitioners

#### Management of mastocytosis

Treatment depends on the specific type of mastocytosis and the severity of symptoms resulting from mast cell activation. The novel treatment involving the TKI avapritinib received European marketing authorization (EMA) for use in ISM in December 2023.

The treatment of mastocytosis aims at mitigating symptoms related to the release of mast cell mediators and tissue infiltration by pathological mast cells. Treatment varies according to whether the disease is cutaneous or systemic and based on the degree of disability experienced by the patient.

This symptomatic treatment may be associated with systemic treatment, which we will detail later (see § 2.3).

## General precautions

Some precautions are advised to avoid outbreaks of mast cell degranulation or, even more seriously, anaphylaxis. These precautions are neither strict nor exhaustive and must be adapted on a case-by-case basis. Particular attention must be paid to the triggering factors. Thus, it is useful to avoid combinations of several factors that can promote mast cell degranulation, such as certain foods and medications, intense physical exercise and sudden thermal variations, where these factors have, in the past, been the cause of an episode of mast cell degranulation in the patient. In the event of confirmed previous anaphylactic shock, an intramuscular self-injectable adrenaline may be used in case of emergency up to a maximum of 2 times: patients must carry an epinephrine pen in duplicate and this can be stored at room temperature. The dose to be used is 0.3 mg if the weight is < 60 kg or 0.5 mg if it is > 60 kg, with 1 injection repeated every 15 min if necessary (up to a maximum of two injections).

All types of medication can be sources of anaphylactic reactions, but a strict contraindication is imposed only in the presence of a previous reaction in a given patient [40].

Planned surgical interventions in adults require precautions, which must be provided to the anesthetist. They are currently well codified: adequate premedication, choice of drugs while avoiding certain muscle blockers, and careful monitoring aimed at early treatment of any hypotension. Anesthetic recommendations have been published by Dewachter P et al. and by Inger Femke Astra Bocca-Tjeertes and Annick A. J. M. Van de Ven [41, 42].

## Symptomatic treatment

This therapy is essentially symptomatic and adaptable to each patient, even if a certain consensus emerges (Table S8).

Second-generation anti-H1 antihistamines (off-label) (levocetirizine, desloratadine, ebastine, etc.) sometimes associated with H2 antihistamines (off-label) (famotidine, cimetidine, etc.) are the key agents used as first-line treatments to block receptors since various cells (endothelial, bronchial, neurological, digestive, etc.) are activated by histamine, which is released by mast cells. Second-generation H1 blockers (non-sedative) act mainly on flushing and pruritus; the effect of H2 blockers is more marked on gastroduodenal manifestations (ulcer type, gastritis, diarrhea). They can be combined to potentiate their effects, particularly on flushing and digestive disorders.

The use of a proton pump inhibitor (off-label) may be proposed when there is partial efficacy of H2 blockers.

Leukotriene inhibitors (montelukast) (off-label) can act not only on pruritus and vasomotor flare-ups but also in cases of urinary or digestive problems.

A mast cell membrane stabilizer (disodium cromoglycate) (off-label) is available only as a compounded preparation. At a dose of 100 or 200 mg 2 or 4 times a day, after 2 to 3 weeks of use this drug allows a reduction in digestive symptoms and, in certain cases, alleviation of pruritus and other systemic symptoms.

The use of an adrenaline pen (self-injectable epinephrine that the patient must carry) is suggested for patients with a history of severe or very severe anaphylactic reactions with anaphylactic shock.

General corticosteroid therapy only has a suspensive effect, sometimes with a risk of rebound on discontinuation and potential deleterious bone risk in this area. It should be avoided in the middle or long term. However, intestinal disintegration corticosteroid therapy, such as budesonide (off-label), can be offered for symptomatic digestive disorders with good efficacy while limiting the adverse effects of general corticosteroid therapy.

Guideline dedicated to specialists.

## Initial assessment

The initial clinical examination must investigate for the presence of elements necessary for the diagnosis of mastocytosis, as well as screening for comorbidities.

### Main goals

- Confirm the diagnosis of mastocytosis;
- Identify any severity factors and possible comorbidities (Table S9);
- Specify the type of initial damage (cutaneous/systemic) and, if present, the extent of skin damage (in terms of BSA [body surface area]) and of the indolent SM;
- Evaluate the impact on QoL (MC-QoL score [43] (Table S10) and DLQI [44]);
- Evaluate the disability linked to mastocytosis: BURDEN_Masto [45], AFIRMM [46] and ISM_ASF scores [47];

• Evaluate the risk of progression of the disease (Cf § 3);

• Establish therapeutic indications.

### Professionals involved

The treatment plan for patients presenting mastocytosis is under the responsibility of doctors specializing in mast cell diseases (dermatologists, hematologists, internists, rheumatologists, and allergists), namely hospital specialists belonging to an expert center, a reference center or their networks of correspondents.

Other health professionals who may be involved in treatment are as follows:

- The referring treating physician;
- Any other specialist whose opinion is necessary depending on the clinical picture (gastroenterologist, cardiologist, urologist, neurologist or psychiatrist);
- The pharmacist: compounding/dispensing medications, assessing the risk of drug interactions;
- A private or hospital nurse in the event of the use of subcutaneous injections and laboratory samples and/or hypnosis;
- A dietician;
- An osteopath;
- A psychologist;
- A social worker.

All these staff must be trained in pathology.

### Interviewing patients

The following must be recorded:

- The patient’s personal surgical and medical history (vertebral fractures, osteoporosis of unspecified etiology and/or low-energy fractures), allergy status (in particular, history of anaphylactic reaction), and hematological abnormatities are used to guide the classification of mastocytosis as nonadvanced or advanced.
- Family history of mastocytosis or signs of mast cell activation.
- General signs (weight/performance status according to the WHO, Karnofsky index) [48] (Table S11).

The patient must be questioned about the possible desire of becoming pregnant or the use of contraception (particularly if teratogenic treatment is being considered).

The impact on quality of life, psychological state (depression assessed with the Hamilton scale [49], anxiety), and cognitive state (problems of concentration, attention, memory and sleep) must be assessed.

Regarding anaphylactic shock, any Hymenoptera or insect stings, medications, foods or situations that have caused signs of anaphylaxis in various forms must be specified: urticarial eruption, exanthema, angioedema, malaise, hypotension and shock, cardiac arrest, dyspnea and bronchospasm, and digestive disorders. It will be necessary to systematically search for anaphylaxis associated with Hymenoptera stings (e.g., wasps, bees, hornets or even certain species of ants) or other insects, general anesthetics, and foods as well as the cofactors present, such as these reactions (hot/cold temperature variation, alcohol, NSAIDs, aspirin, physical effort, stress, etc.). In the event of a reaction described by the patient, the opinion of an allergologist, which may result in allergological tests (prick tests, intradermal reactions, sometimes reintroduction tests), must be sought to differentiate a true allergy from an intolerance, explained by mast cell activation, with the aim of determining appropriate measures to remove the source.

### Clinical symptoms

The different symptoms and their date of appearance must be specified:

- Skin pruritus with or without flushing, urticaria, and angioedema
- Malaise, syncope
- Asthenia
- Digestive discomfort (diarrhea, abdominal pain, peptic ulcer)
- Neuro-psychological manifestations
- Osteoarticular manifestations.

The presence of functional signs may correspond to symptoms of mast cell activation (Table S4). The presence of functional signs affecting a single organ can lead to other differential diagnoses (Table S12). The presence of symptoms of mast cell activation involving at least two organs that evolve in a recurrent and chronic manner must, in fact, prompt a search for systemic mastocytosis without skin lesions. The differential diagnosis is mast cell activation syndrome [50, 51] (Table S12) and possibly hereditary alpha-tryptasemia (Figure S1) [52]. It is important to note that idiopathic mast cell activation syndrome is closely associated with hereditary alpha-tryptasemia, so a form named combined MCAS. Similarly, clonal mast cell activation syndrome is linked to bone marrow mast cell accumulation, a finding that is clinically significant in the context of mast cell activation symptoms.

In patients without skin lesions and who are anaphylactic without a clearly identified allergen, we can assess the risk of presence of clonal mast cell disease, including systemic mastocytosis, using the REMA score, which has a sensitivity of 86% and a specificity of 94% [53]. This score takes the relevant tryptase threshold as < 15 ng/mL or > 25 ng/mL, and considers the presence of clinical symptoms (urticaria, malaise or syncope) as well as gender. Two other scores are defined for this type of patient: the modified REMA score, which utilizes the same clinical and gender criteria but employs a tryptase threshold of 11.4 to 20 ng/ml, with better sensitivity (93%) and specificity (94%) than the REMA score [54], and the NICAS score, which takes into account clinical symptoms and gender, with a threshold for serum tryptase of > 11.4 ng/ml and identification or not of the *KIT* mutation in the blood [55]. Its sensitivity is 75% and its specificity is 100% [55]. Indeed, recently published data indicate that monoclonal mast cell disease is present in 12.7% (28/220) of patients with a history of grade 3 and 4 anaphylaxis after Hymenoptera stings, even if the serum tryptase level is within the normal limit (< 11.4 ng/ml). Among all patients included (374), 93.9% (351 patients) had a normal serum tryptase level, and 8% (28/351) had clonal mast cell disease. Among all patients included (374 patients), clonal mast cell disease was identified in 34 patients (9%). Among these 34 patients, only 5 patients agreed to be assessed using a bone marrow biopsy to screen for SM. All 5 patients had confirmed SMs [56].

SM should be considered in patients with spontaneous vertebral fracture(s) or even after low-energy trauma and/or densitometric osteoporosis without any other identified risk factors, particularly where the basal serum tryptase (ST) level is > 10 ng/L, and/or in the presence of signs/symptoms of mast cell activation (anaphylaxis, digestive signs, etc.).

### Clinical examination

The clinical examination will seek to identify elements enabling diagnosis of mastocytosis, with or without skin lesions, as specified in Table 1 of the NPCD.

In the event of diagnostic confirmation of skin involvement, screening for SM is necessary regardless of the ST level. SM is present in 27% of patients with an ST level < 20 ng/mL and > 11.3 ng/mL and in 15% of patients with an ST level < 11.3 ng/mL [57].

Table S11 contains the differential diagnoses of the various phenotypes of cutaneous mastocytosis.

### Imaging tests

Magnetic resonance imaging (MRI) of the spine can be useful for identifying spinal cord mast cell infiltration. Notably, Riffel P et al. [58] recently published results concerning the ability of spine MRI to identify this infiltrate. In this study, bone marrow infiltration was seen on MRI in 96% of patients with an advanced form of mastocytosis. Bone marrow infiltration is much less common in patients with ISM, including patients with bone involvement. In any case, these examinations must be repeated in the event of recurrent fractures, despite treatment with bisphosphonates, and may lead, in the event of significant spinal cord infiltration, to initiation of cytoreductive treatment.

Bone densitometry was performed at the level of the lumbar spine (L2-L4), the left total hip, and the left femoral neck. The following tests may be considered for further investigation:

- X-rays of the dorsal and lumbar spine and long bones or a low-intensity bone scan
- A thorax-abdomen-pelvis (TAP) scan to investigate for deep lymphadenopathy, hepatosplenomegaly and bone windows for analysis of bone structure
- A brain MRI in the event of neurological signs

### Differentiating cutaneous mastocytosis from systemic mastocytosis

The WHO 2022 criteria and the ICC 2022 diagnostic criteria enable the classification of mastocytosis and the confirmation or denial of a diagnosis of systemic involvement (Table S5). These criteria require the following:

- An osteomedullary biopsy and a myelogram;
- Immunohistochemical analysis or bone marrow mast cell phenotyping was performed to identify pathological CD2 + , CD25 + , and CD30 + mast cells (CD117);
- Investigation for a *KIT*-activating mutation in the blood, bone marrow or other internal organs (apart from the skin);
- Determination of the basal serum tryptase level in search of a value ≥ 20 ng/mL.

If all these criteria are negative, we may confirm CM, and if one or two criteria of clonality are positive (without major criterion) we may confirm a CM with primary MCAS.

### Confirming systemic mastocytosis without skin lesions

Approximately 20% of SMs do not present any skin involvement. These forms are mostly localized to bone marrow alone since 90% are not associated with any other organ damage. They are named BMM and nowadays form a separate entity in the WHO classification but not in the ICC classification [59].

At the same time as the search for systemic damage, additional criteria must be sought to differentiate BMM from the smoldering form, which is characterized by two B-findings (borderline benign) (Table S13) [11], and especially from the advanced SM variants. Indeed, so-called advanced mastocytoses constitute the aggressive form and have at least one C-finding (Consider Cytoreduction) (Table S14) [60], SM associated with an associated hematologic neoplasm (SM-AHN), and mast cell leukemia [61].

### Other immunopathological examinations

Tissue biopsies from other organs, such as the gastrointestinal tract, liver and/or lymph nodes, can also confirm systemic involvement when they reveal an abnormal mast cell infiltrate (according to WHO and ICC 2022 criteria).

Notably, not all anatomopathological, immunological or molecular biology laboratories have technical facilities allowing these routine analyses. In this case, the samples can be sent to the laboratories attached to the reference/competence centers, which have these techniques available.

## Patient care/treatment

### Goals

Controlling the symptoms of mast cell activation is the essential objective to minimize the adverse effects of treatments as much as possible.

The treatment aims are as follows:

- Control symptoms of mast cell activation and regression of functional impairment linked to the disease;
- Improve the quality of life of patients;
- Limit the very frequent adverse effects linked to the prolonged duration of symptomatic treatment.

### Proposal for therapeutic care

Several treatments for mastocytosis have been proposed, in addition to oral symptomatic treatment.

#### Injectable symptomatic treatment

Omalizumab, which can be increased to 300 mg every 2 weeks depending on safety and efficacy, has been shown to be effective in retrospective studies for the management of symptoms of mast cell activation in individuals with indolent mastocytosis at a dose of 150 mg once every 15 days. The first two injections are generally given in a hospital setting [62]. It has been the subject of a systematic review of the literature [63].

#### Nonspecific cytoreductive treatments

Cytoreductive treatments are used empirically.

These treatments are associated with adverse effects and, for the most part, immunosuppression, leading to the prescription of these treatments with caution and in a personalized manner. This prescription must be made after discussion in a multidisciplinary consensus meeting (MCM).

To date, the main treatments that have shown some effectiveness are interferon-alpha [61–63] and 2-chlorodeoxyadenosine (cladribine or 2-CdA)[64–66], but these treatments are rarely prescribed in non-advanced forms of mastocytosis, even if a recent paper on cladribine in these forms of mastocytosis was published in 2024 [67].

Rapamycin may also be of interest in *KIT* D816V + patients and should be the subject of a prescription discussion during the National MCM [64].

#### Tyrosine kinase Inhibitors (TKIs)

To date, imatinib is recognized as ineffective where patients carry the *KIT* D816V mutation in the marrow and/or skin, as was reported in vitro by Zermati et al*.* [65]. On the other hand, this treatment is proposed for SM associated with hypereosinophilic syndromes with the presence of the *FIP1L1*-*PDGFRa* fusion gene and SM without *KIT* mutation at exon 17.

Masitinib (off-label), another KIT inhibitor, is currently undergoing a phase III trial. Two phase II studies have been carried out on variants of mastocytosis associated with disability. The first study [66] showed that disability (composite score) was present in 25 patients (*KIT* gene status negative for 816 mutations) receiving open-label masitinib, with an overall response at 3 months in 56% of them. This result was prolonged into the extension phase. An international phase III study evaluating masitinib vs. a placebo revealed the benefit of masitinib in ISM patients with disability [68]. A 2nd multicenter, international phase III study is currently underway.

Avapritinib (BLU-285) (marketing authorization), an inhibitor of the KIT D816V mutant, is currently being evaluated in the extension (phase II/III PIONEER trial [69]) in indolent systemic forms with disability. In this study, avapritinib reduced uncontrolled symptoms and mast-cell burden in patients with ISM. Moreover, from baseline to Week 24, 76 of 141 patients (54%; 45% to 62%) in the avapritinib group compared to 0/71 patients in the placebo group achieved a 50% reduction in serum tryptase level (*P* < 0.001) [70].This new treatment is the firstTKIs for indolent forms of mastocytosis refractory to symptomatic treatments [71]. In fact, the treatment received marketing authorization in European Union in 2023 but is not yet available for prescription in France.

Another tyrosine kinase inhibitor active against KIT D816V, the compound BLU-263 (elenestinib), is being evaluated in an international phase II/III study in indolent mast cell disease (HARBOR, [72]), in which several European centers are participating. In addition, bezuclastinib, another TKI specific to D816V, is being evaluated in a study (SUMMIT) involving several European centers [73].

Midostaurin is a multikinase inhibitor with Marketing Authorization in France for advanced systemic mastocytosis. There is an open-label phase II study for ISM with interesting efficacy results. However, safety, particularly digestive, is often poor, with adverse effects that include nausea (80% of patients), vomiting (35%) and headaches (10.5%) [74]. Further studies on the use of midostaurin in the treatment of ISM are necessary to determine its future usefulness in the development of therapeutic regimens for this disease.

Table S14 presents the examinations necessary for the implementation of basic treatments for ISM patients with disability and for monitoring treatments.

Table S15 includes precautions to be observed before initiation of treatments such as cladribine or tyrosine kinase inhibitors.

Table S16 presents the examinations necessary for monitoring basic treatments in patients with systemic mastocytosis and disability.

#### Dermatological treatment

PUVA therapy is a classic treatment for cutaneous mastocytosis, especially in the maculopapular form, but this treatment should no longer be offered. Indeed, in a Danish case‒control study, increased prevalence of melanoma was demonstrated in patients with mastocytosis [75]. This intrinsic risk is added to that of phototherapy.

Topical corticosteroids have no place in adults, unlike in children.

A vascular laser or flash lamp could be used to treat telangiectasias of TMEP.

In two publications, a Q-switched pigment laser was reported to have a certain degree of cosmetic efficacy on urticaria pigmentosa spots [76, 77]. A prospective single-center study is underway at CEREMAST, the expert center of Toulouse University Hospital [78].

#### Treatment with calcium and/or vitamin D

In the event of a deficiency in dietary calcium intake (less than 1000 mg/D) and/or in the event of vitamin D deficiency (measured by a level of 25OH D ≤ 30 ng/mL or 75 nmol/L), supplementation is necessary to avoid worsening bone loss. Correcting these deficiencies also makes it possible to improve the efficacy of anti-osteoporotic medications. It is necessary in all cases of mastocytosis.

In the event of calcium intake deficiency, food intake, which is also a source of protein, should be prioritized. To assess whether dietary calcium intake is correct, the patient can be asked to complete the dedicated questionnaire accessible via the following link: CALCIUM—Calcium Calculator|International Osteoporosis Foundation. If food intake is not possible in cases of digestive intolerance, medicinal calcium supplementation is necessary.

In the event of insufficient vitamin D levels, supplementation with vitamin D (D3) in daily or intermittent monthly doses with adaptation of supplementation according to the 25–OH–D level are recommended [79], and therapeutic management is described by Souberbielle et al*.* [80].

#### Rheumatological treatments

Two scenarios must be distinguished: (1) patients with densitometric osteoporosis without an episode of fracture, and (2) patients with severe osteoporosis attributable to mastocytosis, with multiple vertebral fractures (possibly with slightly lowered bone densitometry).

It is likely that in the case of isolated densitometric osteoporosis without fracture, specific treatment is not essential as first-line therapy, and the patient should be evaluated by a rheumatologist [13]. On the other hand, in patients with severe osteoporosis manifested by multiple vertebral fractures, treatment with bisphosphonates rather intravenously must be initiated with the possibility of combining it with tyrosine kinase inhibitors/mast cell activation inhibitor treatment [74, 79].

With respect to denosumab, a major point to consider is the increased risk of vertebral fracture with a fracture cascade upon treatment discontinuation [81]. Indeed, a rebound phenomenon associated with increased markers of bone remodeling, decreased bone mineral density, and the risk of multiple vertebral fractures has been described in postmenopausal osteoporosis patients treated with denosumab [82]. An identical phenomenon is expected in patients treated for osteoporosis associated with mastocytosis. The results of the DENOSUMAST study are awaited within the next 2 years [83].

In addition to pharmacological measures, the correction of risk factors for fracture osteoporosis (smoking, alcohol, etc.), prevention of falls, vitamin-calcium intake, physical activity and a sufficient daily dietary calcium ratio are essential elements of care.

#### Desensitization to hymenoptera venom

In the event of a proven allergy to Hymenoptera venom or possibly a severe or very severe anaphylactic reaction, desensitization or allergen immunotherapy (AIT) is the only protective treatment currently available that has proven effective in the event of a new bite. Some authors recommend life-long continuation of this therapy [84]. AIT may be less protective in patients with severe initial systemic reactions and mastocytosis and/or elevated basal serum tryptase levels (> 11.4 µg/L). Therefore, for safety reasons, it should be prolonged in these patients, although we do not know whether it is imperative to administer it life-long or after what duration of treatment it should be stopped [85].

AIT with Hymenoptera venom is generally well tolerated in patients with mastocytosis [86]. However, cases of poor tolerance to anaphylactic reactions may be observed.

#### Management in cases of poor tolerance to desensitization to hymenoptera venom

Poor tolerance of AIT to Hymenopteran venom with symptoms up to severe anaphylaxis can sometimes lead to the diagnosis of mastocytosis with or without skin lesions if the baseline serum tryptase level is higher than normal [84]. To limit these manifestations linked to desensitization to Hymenoptera venom, the adaptation of tolerance induction protocols seems essential; thus, taking antihistamines, or even treatment with omalizumab (anti-IgE), can be associated with desensitization to Hymenoptera venom to establish protective AIT [62, 63]. This may improve tolerance to desensitization to Hymenoptera venom but at a greater cost, which must be considered during reflection.

#### Vaccinations

In principle there are no vaccine contraindications in the context of mastocytosis, and the vaccination schedule must be adhered to in both children and adults. True allergy to eggs and/or chicken remains a contraindication for certain vaccines and will require advice from a specialized vaccination center. Concerning the vaccine against the SARS-CoV-2 virus, international recommendations [87] specify that, as in the general population, there is no reason to exclude an mRNA vaccine in the absence of confirmed allergy to PEG or polysorbates.

Other precautions in adults include the contraindication of live vaccines (BCG, oral polio, measles, rubella, mumps, yellow fever, and Japanese encephalitis) in the event of background treatment based on cladribine (2-CdA) for at least 18 months after stopping treatment due to lymphopenia induced by the treatment, which may limit antibody response or promote an infectious risk for live vaccines. In this therapeutic context, if such a vaccine is necessary, advice should be sought from an infectious disease specialist.

### Proposed therapeutic management according to the different forms of mastocytosis covered in this NPCD

#### Cutaneous mastocytosis

This is defined by the presence of histologically confirmed cutaneous mastocytosis lesions in adults and the absence of any diagnostic criteria for systemic mastocytosis (Table S5).

##### CM with few symptoms of mast cell activation (isolated skin lesions with few symptoms)

- Avoidance of triggering factors (food, medication, or the environment where feasible);
- Symptomatic treatment;
- No cytoreductive treatment;
- No phototherapy due to the increased risk of melanoma in patients with mastocytosis [75];
- Vascular laser treatment for telangiectatic and/or pigmentary lesions in affected areas should be considered if DLQI score [44] and CM-QoL score [43] are strongly affected;
- Correction of calcium intake deficiencies and vitamin D supplementation if values are suboptimal.

##### *CM with disabling symptoms of mast cell activation *[43–47]

- Avoidance of triggering factors (food, medication, or the environment where feasible);
- Symptomatic treatment (Table S8) with anti-H1, anti-H2, mast cell membrane stabilizers and antileukotrienes (montelukast 10 mg/day);
- If the first line of symptomatic treatment fails, discuss treatment with omalizumab (see § 2.2 of the guide for treating physicians);
- If symptomatic treatment fails, discuss treatment with peg-interferon (Cf. § 2.2.2. of this guide) or with cladribine (Cf. § 2.2.2. of this guide);
- Avoidance of phototherapy due to increased risk of melanoma in patients with mastocytosis [75];
- Vascular laser treatment for telangiectatic and/or pigmentary lesions in affected areas should be given if the DLQI score [44] and strong skin dimension on the CM-QoL score [43] are altered;
- Correction of calcium intake deficiencies and vitamin D supplementation if values are suboptimal;
- Pain treatment if necessary (discuss with the pain center a treatment targeting the N-methyl-D-aspartate (NMDA) receptor in the event of opioid/fentanyl failure/intolerance) [88];
- Lifelong desensitization of Hymenoptera allergy.

#### Indolent systemic mastocytosis (ISM)

This is defined as follows:

- Presence of the major diagnostic criterion and at least one minor diagnostic criterion or three minor diagnostic criteria of systemic mastocytosis with skin lesions (Table S5);
- Presence of skin lesions associated with mastocytosis or without skin lesions but with serum tryptase > 125 ng/mL;
- Absence of B-findings (Table S13) or C-findings (Table S14), no associated hematological malignancy, and no mast cell leukemia.

##### ISMs with few mast cell activation symptoms and associated cutaneous lesions.

- Avoidance of triggering factors (food, medication, or the environment where feasible);
- Symptomatic treatment;
- No cytoreductive treatment;
- No phototherapy due to increased risk of melanoma in patients with mastocytosis [75];
- Vascular laser treatment for telangiectatic and/or pigmentary lesions in affected areas is recommended if the DLQI score [44] and strong skin dimension on the CM-QoL score [43] are altered;
- Discuss treatment with calcium and/or vitamin D3 if values are suboptimal;
- Anti-osteoporotic bisphosphonate therapy should be used intravenously rather than orally with or without specific TKI therapy in severe osteoporosis (T score <  −2.5) especially in cases of vertebral fractures [79]. If denosumab is prescribed, a rebound effect should be anticipated if the treatment is stopped. The current state of knowledge in the field of postmenopausal osteoporosis consists of prescribing one or more infusions of zoledronic acid with close monitoring of bone resorption markers (serum CTX) [89];
- Lifelong desensitization in the event of severe anaphylaxis after Hymenoptera stings;
- Follow-up for systemic mastocytosis should be ensured (Cf. § 4).

##### *ISM with disabling mast cell activation symptoms *[43–47]

- Avoidance of triggering factors (food, medication, or the environment where feasible);
- Symptomatic treatment (Table S8) with anti-H1, anti-H2, and mast cell membrane stabilizers and/or antileukotrienes (montelukast 10 mg/day);
- In the event of failure of the first line of symptomatic treatment, discuss treatment with omalizumab (see § 2 of the guide for treating physicians) or inclusion in a therapeutic protocol: masitinib, B263;
- If inclusion in a clinical study is impossible and there is failure of or intolerance to omalizumab, discuss treatment with peg-interferon alpha (see § 2.2.2. of this guide) or cladribine (see § 2.2.2. of this guide);
- Avoid phototherapy due to the increased risk of melanoma in patients with mastocytosis [75];
- Vascular laser treatment for telangiectatic and/or pigmentary laser for pigmentary lesions in the affected areas has been used whether the DLQI score [44] and skin dimension on the MC_QoL score were altered (Table S8) [43];
- Discuss treatment with calcium and/or vitamin D3 if values are suboptimal;
- Anti-osteoporotic treatment with bisphosphonates should be used orally rather than intravenously, with or without cytoreductive treatment in the case of severe osteoporosis (T score less than -2.5), especially in cases of vertebral fractures [79]. If denosumab is prescribed, a rebound effect should be anticipated if the treatment is stopped. The current state of knowledge in the field of postmenopausal osteoporosis consists of prescribing one or more infusions of zoledronic acid with close monitoring of markers of bone resorption such as serum CTX;
- Pain treatment if necessary (discuss with the pain management center a treatment targeting the N-methyl-D-aspartate (NMDA) receptor in the event of opioid/fentanyl failure/intolerance) [82];
- Lifelong desensitization in cases of severe anaphylaxis following Hymenoptera stings;
- Follow-up for systemic mastocytosis is necessary (Cf. § 4).

#### Bone marrow mastocytosis (BMM)

This is a new entity in the 2022 WHO classification but not in the 2022 ICC classification).

It is defined as follows:

- Presence of the major diagnostic criterion and at least one minor diagnostic criterion or three minor diagnostic criteria of SM without skin lesions, provided that tryptase is < 125 ng/ml (Table S5);
- Absence of mastocytosis skin lesions;
- Absence of criterion B (Table S13) or C (Table S14), of an associated hematological malignancy and of mast cell leukemia.

These patients most frequently present with a disease caused by bone fracture or anaphylactic shock, and many do not present symptoms of disabling mast cell activation.

##### BMMs with few symptoms of mast cell activation

- Avoidance of triggering factors (food, medication, or environmental factors where feasible);
- Symptomatic treatment;
- No cytoreductive treatment other than in bone fracture or anaphylactic cycle;
- No phototherapy due to increased risk of melanoma in patients with mastocytosis [72];
- Discuss treatment with calcium and/or vitamin D3 if values are suboptimal;
- Discuss anti-osteoporotic treatment with bisphosphonates rather than intravenously with or without cytoreductive treatment, if severe osteoporosis (T score <  −2.5) and especially in cases of vertebral fractures [76]. If denosumab is prescribed, a rebound effect should be anticipated if the treatment is stopped. The present state of knowledge in the domain of postmenopausal osteoporosis is characterized by the prescription of one or more infusions of zoledronic acid, accompanied by meticulous monitoring of bone resorption markers, specifically serum CTX [86];
- Lifelong desensitization in the event of severe anaphylaxis after Hymenoptera stings;
- Ensure follow-up as for systemic mastocytosis (Cf. § 4).

##### BMMs with disabling mast cell activation symptoms [38–42]

- This entity is exceptional.
  Avoid triggering factors (food, medication, or the environment where feasible):
- Symptomatic treatment (Table S8) with anti-H1, anti-H2, mast cell membrane stabilizers and/or antileukotrienes (montelukast 10 mg/day);
- In the event of failure of the first line of symptomatic treatment, discuss treatment with omalizumab (see § 2 of the guide for treating physicians) or inclusion in a therapeutic protocol: masitinib, Blu-263 or other studies;
- If inclusion in a clinical study is not feasible and there is failure or intolerance to omalizumab, treatment with peg-interferon-alpha or cladribine should be discussed;
- Treatment with calcium and/or vitamin D3 should be discussed if values are suboptimal;
- Anti-osteoporotic treatment with oral bisphosphonates rather than intravenous administration, with or without cytoreductive treatment, should be discussed in the event of severe osteoporosis (T score <  −2.5), and especially in cases of vertebral fractures [76]. If denosumab is prescribed, a rebound effect should be anticipated if the treatment is stopped. The current state of knowledge in the field of postmenopausal osteoporosis consists of prescribing one or more infusions of zoledronic acid with close monitoring of markers of bone resorption (serum CTX);
- Pain therapy if necessary (discuss with the pain management center a treatment targeting the N-methyl-D-aspartate (NMDA) receptor in the event of opioid/fentanyl failure/intolerance [79];
- Lifelong desensitization in cases of severe anaphylaxis after Hymenoptera stings;
- Ensure follow-up as for systemic mastocytosis (Cf. § 4).

#### ***Smoldering systemic mastocytosis with or without cutaneous lesions and with or without disabling symptoms of mast cell activation ***[43–47]

These conditions are defined as follows:

- Presence of the major diagnostic criterion and at least one minor diagnostic criterion or three minor diagnostic criteria for systemic mastocytosis (Table S5).
- Presence of two B-findings among the three defined: (1) significant mast cell mass defined by a serum tryptase level > 200 ng/mL and/or more than 30% mast cells identified on osteomedullary biopsy slides and/or a KIT D816V VAF in blood or bone marrow > 10%. This last criterion is noted in the WHO criteria 2022 but not in the ICC criteria 2022. Moreover, while the term “and/or” is found in the WHO criteria 2022, in the ICC criteria 2022 the term “or” is used (2) organomegaly (hepatosplenomegaly, lymphadenopathy (> 1 cm for the ICC criteria 2022) without organopathy and (3) dysmyelopoiesis without cytopenia (Table S13).
- Cytoreductive treatment in general if symptomatic.

#### Other forms of mastocytosis of more recent definition

##### Cutaneous mastocytosis with primary MCAS

These are defined as follows:

- Presence of one or two minor criteria for systemic mastocytosis, at least one of which is clonality (*KIT* D816V + mutation in the marrow/blood or expression of CD25/CD2/CD30 + by mast cells in the marrow);
- Presence of skin lesions associated with mastocytosis;
- Absence of sufficient diagnostic criteria to make a diagnosis of systemic mastocytosis.

##### 8.3.5.1.a Treatment of CM with primary MCAS (abnormal mast cell immunophenotype and/or KIT D816V mutation) and few symptoms of mast cell activation

See ISM with few symptoms’ indicative of mast cell activation (§2.3.2.1).

##### 8.3.5.1.b Treatment of CM with primary MCAS (abnormal mast cell immunophenotype and/or KIT D816V mutation) and disabling symptoms of mast cell activation [43–47]

Treatment of ISM with disabling symptoms of mast cell activation is recommended (§2.3.2.1).

##### Special cases of patients with one to 2 minor WHO criteria (abnormal mast cell immunophenotype and/or *KIT* D816V mutation) and without skin lesions

This is defined as follows:

- Presence of one or two minor criteria for systemic mastocytosis, at least one of which is clonality (*KIT* D816V + in marrow or blood, or expression of CD25/CD2 or CD30 by mast cells);
- Absence of skin lesions associated with mastocytosis;
- Absence of diagnostic criteria for systemic mastocytosis.

This entity borders with monoclonal MCAS (Figure S1) or marrow mast cell accumulation of clinical importance with symptoms of mast cell activation [89].

##### 8.3.5.2.a Patients with one to 2 minor WHO criteria (abnormal mast cell immunophenotype and/or *KIT* D816V mutation) and with few symptoms of mast cell activation, also referred to as primary MCAS or clonal MCAS.

This is a very rare entity that manifests through osteoporotic damage and/or bone fractures. More frequently, this entity is discovered through anaphylactic reactions.

Management is based on the treatment of ISM with few symptoms of mast cell activation.

##### 8.3.5.2.b Patients with one to two minor WHO criteria (abnormal mast cell immunophenotype and/or *KIT* D816V mutation) and disabling symptoms of mast cell activation [43–47]

This disease is characterized by grade 3, 4 or 5 anaphylaxis [90]. The first two grades are not considered anaphylaxis.

Management is based on the treatment of ISM with disabling symptoms of mast cell activation (Cf. § 4).

## Prognosis of the various forms of mastocytosis addressed in this NPCD

Findings from CEREMAST indicate that CM does not generally progress to SM (average follow-up of 10 years) (unpublished data).

The prognosis for patients with CM is better than for patients with ISM [90].

CM with primary MCAS is likely to have a similar course to ISM, but this hypothesis requires confirmation by future studies.

According to a paper published in 2014 for a cohort of 59 patients, almost all patients had SM [91].

In rare cases, the indolent systemic form may progress to an aggressive form. Indeed, the latest European cohort data show that 2.5% of indolent systemic forms progress to advanced forms [92]. This value may be overestimated due to the fact that advanced forms are very likely more frequently reported in the European database than indolent forms. Furthermore, another study by Lim KH et al*.* [93] showed that individuals with ISM had a life expectancy comparable to that of the American population matched for age and sex.

The recent IPSM score (International prognostic scoring system for mastocytosis), constructed using clinical and biological data from a European registry of 1681 patients, includes a section for indolent forms and another for advanced forms [94]. In indolent forms, the factors associated with a poor prognosis for survival are aged ≥ 60 years and with an increase in alkaline phosphatase to a level ≥ 100 IU/L [94].

Furthermore, a recent study revealed that at the molecular level, the presence of an *ASXL-1*, *RUNX1* or *DNMT3A* mutation with an allele frequency ≥ 30% was associated with decreased overall survival in patients with ISM [95]. However, screening for these mutations is not carried out routinely in ISM in the absence of associated hematological abnormalities.

Unlike mastocytosis in children, which most often regresses spontaneously, mastocytosis in adults remains chronic despite a few reports of spontaneous regression [96]. Urticaria pigmentosa may regress over time: a 2002 study revealed regression of UP lesions in 10% of patients within a cohort of 106 patients with adult-onset mastocytosis followed for 10 years [97].

Patients with BMM probably have the same course as those with ISM, except for a greater risk of anaphylactic shock and/or bone fractures.

Patients presenting smoldering-type SM frequently experience a diminution in their quality of life and little change in their vital prognosis [97, 98].

## Patient monitoring

Mastocytosis is a chronic disease that is disabling to a greater or lesser degree throughout life, necessitating long-term treatment. Treatment monitoring is carried out jointly by the mastocytosis specialist and the referring general practitioner.

### Goals

- Evaluate the efficacy and safety of treatments;
- Plan to gradually taper treatment and shorten the duration of maintenance therapy;
- Cary out serum tryptase and albumin assay, complete blood count (CBC) and liver tests at least once a year to assess the absence of progression of the indolent form of mastocytosis toward a more advanced form;
- Assess bone mineral density every 24 to 36 months to verify the absence of loss of bone density (for patients with ISM, BMM or even CM with primary MCAS).
- Evaluate spinal cord mast cell infiltrate at least at the beginning of the disease by spinal MRI in accordance with the recent publication by Riffel P et al*.* [58] (this examination is not performed routinely at all expert centers).

#### 10.1.1 Short-term follow-up—Initial goal = evaluation of symptoms of mast cell activation

#### a Definition

Symptoms should be evaluated after 3 to 6 months of treatment to:

- Verify the absence of any new symptoms;
- Check for symptoms of mast cell activation.

The course is generally slow and favorable, and it often takes 3–6 months to achieve partial or complete control of symptoms.

#### 10.1.1.b Action to be taken in the absence of a 3-monthly evaluation according to expert consensus and specialist advice (competence center or reference center)

#### 10.1.1.c What to do after the initial evaluation

It is recommended that a reduction in symptomatic treatment be implemented in order to ascertain the minimum effective dose.

#### 10.1.1.d What to do in the event of a relapse.

Definition: relapse comprises the appearance of at least one symptom of mast cell activation for 1 month that does not resolve spontaneously within 1 week.

The proposed treatment for relapse in patients who have received symptomatic treatment comprises experimental treatment used in a clinical study such as the masitinib, HARBOR, or other studies. The implementation of these treatments must be discussed with an expert center for mastocytosis.

(!) Mistakes to avoid.

One mistake to avoid is to stipulate complete and rapid control (within 3 months) of symptoms of mast cell activation (risk of undue therapeutic escalation).

### Rhythm and content of consultations

The frequency of consultations and performance of additional examinations must be adapted to:

- The patient’s clinical condition;
- The severity and course of symptoms of mast cell activation under treatment;
- The treatments used (monitoring, safety, adverse effects).

Evaluation of treatment efficacy is primarily clinical, with monitoring at least every 3 months until the disease symptoms are completely under control.

#### Clinical examination

The follow-up clinical examination is identical to that carried out during the initial evaluation and must aim to determine whether:

- The symptoms of mast cell activation are well controlled
- There are adverse effects associated with the treatment, such as weight gain, drowsiness, fatigue, etc.

#### Monitoring mastocytosis activity

Monitoring entails the assessment of serum tryptase levels at 6–12 months intervals, contingent upon the frequency of symptoms and overall disability, with subsequent annual evaluations. This monitoring is associated with complete blood count (CBC), platelet count, liver function test, and serum albumin measurement. In the event of an isolated, significant increase in serum tryptase over the patient's baseline value and confirmed by a second sample, it is essential to inquire about the potential worsening of the specific form of mastocytosis. In the event of new bone pain, blood tests are indicated to check the CBC, perform an LFT, and determine albumin levels, which, together with abdominal ultrasound and X-rays can rule out any worsening of mastocytosis.

In the event of abnormalities in the CBC, platelets, or liver tests, or of a decrease in serum ALB, a further bone marrow assessment and imaging examinations specifically to screen for an advanced form of mastocytosis will be necessary.

### Treatment initiation (Table S15)

Depending on the type of treatment chosen, several examinations will have to be carried out before initiation of therapy.

#### Precautions when starting disease-modifying treatments for mastocytosis (Table S16)

A number of precautionary measures must be observed when initiating treatment with tyrosine kinase inhibitors.

#### Treatment monitoring (Table S17)

The monitoring methods for the various treatments are detailed in Table S17.

#### Stopping treatment

Symptomatic treatment is continued in the long term.

The duration of treatment with tyrosine kinase inhibitors (avapritinib, masitinib, elenestenib, bezuclastinib, midostaurin, etc.) has not yet been established.

### Possible disabilities

Mastocytosis may be a source of handicap (feeling of fatigue, physical and psychological exhaustion, osteoarticular pain, memory problems, anxiety and insomnia, etc.) [43], but treatment can also produce adverse effects.

## Patient information

Patients and/or their families should be informed of their form of mastocytosis, its prognosis, treatments and possible adverse effects, and examinations should be carried out during follow-up to monitor disease activity and detect possible complications. Patients should also be informed of the existence of referral/competence centers for their disease.

Moreover, patients should be informed of the existence of patient associations: TMS, The Mast Cell Society (Home—TMS—The Mast Cell Disease Society, Inc (tmsforacure.org)); ASSOMAST (Association of patients with mastocytosis or MCAS: https://assomast.org) and AFIRMM (Association of patients and for research in mast cell pathologies: http://www.afirmm.com).

Finally, the AIM (About—The American Initiative in Mast Cell Diseases (aimcd.net) and the ECNM (European Competence Network on Mastocytosis | ECNM 2024 (ecnm-conference.com) [99] can also provide information.

## Conclusion

We present practical recommendations for both the step-by-step diagnostic workup of mastocytosis and the management and follow-up of the full spectrum of nonadvanced mastocytosis in adulthood.

Didactic prescription summaries are also provided, with the aim of facilitating the prescription of mast cell-targeted drugs, along with practical diagnostic and therapeutic algorithms. The present expert consensus is expected to inform and assist health professionals in the diagnostic and/or therapeutic management of patients with nonadvanced mastocytosis.

## Supplementary Information

Supplementary material 1

## Data Availability

Not applicable.

## Abbreviations

ASO qPCR: Allele-specific oligonucleotide quantitative polymerase chain reaction
Beta hCG: Beta subunit of human chorionic gonadotropin (hCG)
BMD: Bone mineral density
CBC: Complete blood count
CD: Cluster of differentiation
NPCD: National protocol for care and diagnosis
CEREMAST: French national expert center for mastocytoses
MIS: Mastocytosis in skin
CM: Cutaneous mastocytosis
TMEP: Telangiectasia macularis eruptiva perstans
SM: Systemic mastocytosis
BMM: Bone marrow mastocytosis
SSM: Smoldering (slowly progressive, “borderline”) systemic mastocytosis
DLQI: Dermatology life quality index
IgE: Immunoglobulin E
ISM: Indolent systemic mastocytosis
MCAS: Mast cell activation syndrome
MA: Marketing authorization
MARIH: Filiere de sante maladies rares immuno-hématologiques (French network for rare immuno-hematological diseases)
MCM: Multidisciplinary consensus meeting
MRI: Magnetic resonance imaging
NSAIDs: Nonsteroidal anti-inflammatory drugs
PUVA: Psoralen and UVA
QoL: Quality of life
ST: Serum tryptase
TSH: Thyroid-stimulating hormone
TKIs: Tyrosine kinase inhibitors
WHO: World Health Organization

## Declarations

### Competing of interests

None declared.
