# Supplementary material for: French guidelines for the management of nonadvanced mastocytosis in adults
Source: Orphanet J Rare Dis. 2025 Oct 2;20:499. doi: 10.1186/s13023-025-03764-7 (PMC12492810; doi:10.1186/s13023-025-03764-7)

# Supplemental Information

**Table S1: Multidisciplinary expert working group**

| Coordinator |
| --- |
| Dr Cristina Livideanu, dermatologist, Toulouse |
| Leading group; editorial group |
| Prof. Michel Arock, medical biologist, Paris  Dr Stéphane Barete, dermatologist, Paris  Prof. Ghandi Damaj, hematologist, Caen  Prof. Olivier Hermine, hematologist, Paris  Dr Julien Rossignol, hematologist, Paris |
| Rating group; reading group |
| Prof. Karine Briot, rheumatologist, Paris  Dr Quentin Cabrera, hematologist, Réunion  Dr Yannick Degboé, rheumatologist, Toulouse  Dr Laurent Frenzel, hematologist, Paris  Prof. Caroline Gaudy, dermatologist, Marseille  Dr Clément Gourguechon, hematologist, Amiens  Prof. Laurent Guilleminault, allergologist, Toulouse  Prof. Mohamed Hamidou, internist, Nantes  Dr Cyrille Hoarau, allergologist, Tours  Dr Rose-Marie Javier, rheumatologist, Strasbourg  Prof. David Launay, internist, Lille  Dr Edwige Le Moeul, internist, Rennes  Dr Claire Mailhol, allergologist, Toulouse  Prof. Carle Paul, dermatologist, Toulouse  Prof. Angèle Soria, dermato-allergologist, Paris  Dr Ewa Wierzbicka Hainaut, dermatologist, Poitiers |

**Table S2:** **Differential diagnosis of cutaneous mastocytosis**

| Type of cutaneous mastocytosis | Various differential diagnoses (non-exhaustive list) |
| --- | --- |
| Pigmentary maculopapular mastocytosis (urticaria pigmentosa) | Lesions possibly associated with dermographism +/- associated pruritus:   - Folliculitis of the trunk or thighs with residual postinflammatory pigmentary lesions - Pigmentogenic lichen planus - Amyloidosis - Syringomas - Actinic lentigos - Cutaneous plasmacytoma   Any pigmented lesion associated with epidermal damage:   - Seborrheic keratoses - Porokeratoses |
| Erythematous maculopapular mastocytosis | Lesions possibly associated with dermographism +/- pruritus:   - Lichen planus - Multiples syringomas |
| Mastocytoma (exceptional in adults) | Juvenile xanthogranuloma/cutaneous histiocytosis |
| Diffuse cutaneous mastocytosis  (exceptional in adults) | Any cause of erythroderma, in some cases sclerodermiform without signs of epidermal damage |

**Table S3: Scoring system for assessing the risk of developing SM in patients with cutaneous mastocytosis** [32]

| Item | Score |
| --- | --- |
| Tryptase (ng/ml) |  |
| < 10.0 | -1 |
| ≥ 10 < 15 | 0 |
| ≥15 < 20 | 1 |
| ≥ 20 | 3 |
| Osteoporosis or osteoarticular pain | 1 |
| Cardiovascular or constitutional symptoms | 1 |

Score of -1 or 0: low risk of SM (≤ 25%).

Score of 1 or 2: moderate risk of SM (40–70%).

Score of 3, 4 or 5: high risk of SM (≥ 86%)

**Table S4: Symptoms of mast cell activation** [50]

| Affected Organ | Symptoms/signs |
| --- | --- |
| Skin | Pruritus |
|  | Flush |
|  | Urticaria/angioedema |
| Gastrointestinal tract | Abdominal pain |
|  | Diarrhea |
| Respiratory system and mucous membranes | Nasal congestion/Rhinorrhea |
|  | Sneeze |
|  | Laryngeal edema |
|  | Wheezing |
|  | Bronchoconstriction |
| Cardiovascular system | Faintness |
|  | Presyncope, collapse |
|  | Hypotension, tachycardia |

*Patients with mastocytosis may present many more symptoms of mast cell activation, such as urinary frequency with nocturia, headache, brain fog, depression, anxiety, memory problems, concentration problems, sleep problems, anaphylaxis, fatigue, fever, cachexia, etc. [49].

**Table S5: Diagnostic criteria for SM according to WHO 2022** [33]

| Diagnostic criteria | Biological abnormality |
| --- | --- |
| Major criteria | Presence of multifocal dense infiltrates of ≥15 aggregated mast cells in bone marrow biopsy and/or in other extracutaneous tissues |
| Minor criteria | 1. On bone marrow smear or bone marrow biopsy: more than 25% of the mast cells in the infiltrate are spindle-shaped or of atypical morphology, with a bi- or multi-lobed nucleus |
|  | 1. Abnormal expression of CD2, CD25, and/or CD30 by mast cells in bone marrow or other extracutaneous organs |
|  | 1. Presence of an activating mutation of the KIT gene |
|  | 1. Serum tryptase level > 20 ng/mL (not applicable in the event of other associated myeloid neoplasm and to be adjusted if hereditary alpha-tryptasemia is present); this latter notion is described in WHO Classification 2022 but not in ICC Classification 2022 |

A diagnosis of SM is made in the presence of:

1. The major criterion and at least one minor criterion, or;

2. Three minor criteria.

**Table S6: Differences between diagnostic criteria in WHO 2022 and ICC 2022**

| **WHO classification 2022** | **ICC classification 2022** |
| --- | --- |
| **1) SM = presence of major criterion + 1 minor criterion or of three minor criteria**  2) The final minor criterion (serum tryptase level) must be adjusted if other associated myeloid neoplasm and hereditary alpha-tryptasemia present | 1. **SM = presence of major criterion or of**   **three minor criteria**  2) The final minor criterion (serum tryptase level) must be adjusted if other associated myeloid neoplasm present |
| **Smoldering SM = 2 B-findings among**   1. **Large mast cell mass**  - At bone marrow biopsy > 30% of cells are mast cells grouped or not in aggregates, and/or the serum tryptase level is > 200 µg/L, and/or VAF KIT in the blood or bone marrow >10%  1. **Bone marrow dysplasia**  - Signs of dysplasia of other hematopoietic lineages but without diagnostic arguments in favor of another hematologic malignancy (normal CBC)  1. **Organomegaly**  - Hepatosplenomegaly without organ abnormality and/or lymphadenopathy | **Smoldering SM = 2 B-findings among**   1. **Large mast cell mass**  - At bone marrow biopsy > 30% of cells are mast cells grouped or not in aggregates, and/or the serum tryptase level is > 200 µg/L  1. **Bone marrow dysplasia**  - Signs of dysplasia of other hematopoietic lineages but without diagnostic arguments in favor of another hematologic malignancy (normal CBC)   **3) Organomegaly**   - Hepatosplenomegaly without organ abnormality and/or lymphadenopathy > 1 cm |

**Table S7: Differences in mastocytosis classification between WHO classification 2022 and ICC classification 2022**

| **WHO classification 2022** | **ICC classification 2022** |
| --- | --- |
| Cutaneous mastocytosis   - Maculopapular mastocytosis - monomorphic - polymorphic - Diffuse cutaneous mastocytosis - Cutaneous mastocytoma - Solitary - Multiple | Cutaneous mastocytosis   - Maculopapular mastocytosis - Diffuse cutaneous mastocytosis - Cutaneous mastocytoma |
| SM are classified as:   - Indolent SM - Bone marrow mastocytosis (BMM) - Smoldering SM - Advanced SM | SM are classified as:   - Indolent SM, including BMM - Smoldering SM - Advanced SM |

**Table S8: Symptom****atic treatments**

| **Treatments** | **Indications** | **Route** |
| --- | --- | --- |
| H1 antihistamine (desloratadine, levocetirizine, ebastine, fexofenadine and others) | Cutaneous mcas* | Oral, continuous |
| H2 Antihistamine (cimetidine/famotidine)  Not available as specialty but available in master preparation | 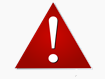Anti-H1 resistant digestive + /- cutaneous MCAS | Oral, continuous |
| Proton pump inhibitor | Anti-H1 resistant digestive mcas | Oral, if necessary, limited time? |
| Mast cell membrane stabilizers (sodium cromoglycate: Not available as specialty but available in master preparation) | Resistance to previous treatment cutaneous or digestive mcas  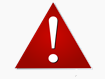 | Oral, continuously |
| Antileukotrienes (montelukast) | Resistance to previous treatment cutaneous mcas  Urinary mcas  Respiratory mcas | Oral, continuously |

*mcas: Mast cell activation symptoms

**Table S9: Severity factors and possible comorbidities**

| Severity factors |
| --- |
| 1) possibility of presenting severe anaphylactic reactions (grade 3 anaphylaxis) or cardiac arrest (grade 4 anaphylaxis), allergic or without any identified allergen linked to massive mast cell degranulation.  2) presence of bone fractures with or without osteoporosis on densitometric examination. |
| Comorbidities |
| The main comorbidity of SM is the presence of osteoporosis on densitometric examination [14], which exposes the patient to the risk of bone fracture. |

In some cases, digestive, constitutional or cardiovascular symptoms, whether are very serious, may be sources of disability for patients

**Table S10: Mastocytosis Quality of Life Questionnaire (Figure S2 after Siebenhaar et al, 2016** [43]**)**

|  | **None** | **Some-what** | **Moderately** | **Very** | **Very Much** |
| --- | --- | --- | --- | --- | --- |
| How severely were you affected by the following symptoms in **the last 2 weeks**? |  |  |  |  |  |
| 1. Itching | ❏ | ❏ | ❏ | ❏ | ❏ |
| 1. Skin redness/swelling | ❏ | ❏ | ❏ | ❏ | ❏ |
| 1. Sudden feeling of warmth and reddening of the face (flush episodes) | ❏ | ❏ | ❏ | ❏ | ❏ |
| 1. Diarrhea/loose stools | ❏ | ❏ | ❏ | ❏ | ❏ |
| 1. Fatigue/exhaustion | ❏ | ❏ | ❏ | ❏ | ❏ |
| 1. Headache | ❏ | ❏ | ❏ | ❏ | ❏ |
| 1. Muscle or joint pain | ❏ | ❏ | ❏ | ❏ | ❏ |
| 1. Difficulty concentrating | ❏ | ❏ | ❏ | ❏ | ❏ |
|  | **None** | **Some-what** | **Moderately** | **Very** | **Very Much** |
| Please indicate how often were you hampered in your daily life in the following areas during the **past 2 weeks** as a result of your mastocytosis. |  |  |  |  |  |
| 1. School/University/Work | ❏ | ❏ | ❏ | ❏ | ❏ |
| 1. Sport/Physical Activity | ❏ | ❏ | ❏ | ❏ | ❏ |
| 1. Sleep | ❏ | ❏ | ❏ | ❏ | ❏ |
| 1. Sexual activity | ❏ | ❏ | ❏ | ❏ | ❏ |
| 1. Leisure time | ❏ | ❏ | ❏ | ❏ | ❏ |
| 1. Relationships (friends, family, partner, coworkers) | ❏ | ❏ | ❏ | ❏ | ❏ |

|  | **Never** | **Seldom** | **Occasionally** | **Often** | **Very often** |
| --- | --- | --- | --- | --- | --- |
| We would like to further study difficulties and problems that may be associated with your mastocytosis using the following questions. Please answer according to your experiences over the **past 2 weeks**. |  |  |  |  |  |
| 1. In the past 2 weeks, were you tired during the day because you did not sleep well the night before? | ❏ | ❏ | ❏ | ❏ | ❏ |
| 1. In the past 2 weeks, did you have to change your choice of food and drinks due to your mastocytosis? | ❏ | ❏ | ❏ | ❏ | ❏ |
| 1. Did you feel less capable in the past 2 weeks due to your mastocytosis? | ❏ | ❏ | ❏ | ❏ | ❏ |

|  | **Never** | **Seldom** | **Occasionally** | **Often** | **Very often** |
| --- | --- | --- | --- | --- | --- |
| 1. Have you been burdened by the symptoms of your mastocytosis in the past 2 weeks? | ❏ | ❏ | ❏ | ❏ | ❏ |
| 1. Has your choice of what to wear been restricted in the past 2 weeks due to mastocytosis? | ❏ | ❏ | ❏ | ❏ | ❏ |
| 1. In the past 2 weeks, were you ever afraid you might suffer an allergic reaction due to mastocytosis? | ❏ | ❏ | ❏ | ❏ | ❏ |
| 1. In the past 2 weeks, were you ever afraid that you might receive the wrong treatment if you became unconscious or suffered an accident due to your mastocytosis? | ❏ | ❏ | ❏ | ❏ | ❏ |
| 1. Did you feel uncomfortable in public at any time during the past 2 weeks due to mastocytosis? | ❏ | ❏ | ❏ | ❏ | ❏ |
| 1. In the past 2 weeks, were you afraid of further worsening of your mastocytosis? | ❏ | ❏ | ❏ | ❏ | ❏ |

|  | **Never** | **Seldom** | **Occasionally** | **Often** | **Very often** |
| --- | --- | --- | --- | --- | --- |
| In the **past 2 weeks** did you feel |  |  |  |  |  |
| 1. ...a lack of motivation? | ❏ | ❏ | ❏ | ❏ | ❏ |
| 1. ...alone with your illness? | ❏ | ❏ | ❏ | ❏ | ❏ |
| 1. ...concerned? | ❏ | ❏ | ❏ | ❏ | ❏ |
| 1. ...sad? | ❏ | ❏ | ❏ | ❏ | ❏ |

**Table S11: Karnofsky index** [48]

| Able to carry on normal activity and to work; no special care needed. | 100% | Normal, no complaints; no evidence of disease. |
| --- | --- | --- |
|  | 90% | Able to carry on normal activity; minor signs or symptoms of disease. |
|  | 80% | Normal activity with effort; some signs or symptoms of disease. |
| Unable to work; able to live at home and care for most personal needs; varying amount of assistance needed. | 70% | Cares for self; unable to carry on normal activity or to do active work. |
|  | 60% | Requires occasional assistance but is able to care for most of his personal needs. |
|  | 50% | Requires considerable assistance and frequent  medical care. |
|  | 40% | Disabled; requires special care and assistance. |
| Unable to care for self; requires equivalent of  institutional or hospital care; disease may be  progressing rapidly. | 30% | Severely disabled; hospital admission is indicated although death not imminent. |
|  | 20% | Very sick; hospital admission necessary; active  supportive treatment necessary. |
| Terminal state | 10% | Moribund; fatal processes progressing rapidly. |
|  | 0% | Dead |

**Table S12: Other differential diagnoses in the face of symptoms such as mast cell activation**

| Advanced mast cell diseases |
| --- |
| - Aggressive systemic mastocytosis - Mast cell leukemia - Systemic mastocytosis associated with other blood dyscrasias, according to WHO classification 2022, but only myeloid diseases according to ICC classification 2022 |
| - Endocrine diseases |
| - Adrenal tumors - VIPoma - Gastrinoma - Carcinoid syndrome - Diabetes - Medullary thyroid carcinoma - Estrogen or testosterone deficiencies |
| Gastro-intestinal diseases |
| - Peptic ulcer - *Helicobacter pylori* infection - Ulcerative colitis - Gallbladder lithiasis - Digestive parasitosis - Celiac disease - Digestive allergy |
| Cardiovascular diseases |
| - Heart disease, aortic stenosis - High blood pressure - Vasculitis - Idiopathic anaphylaxis |
| Tumoral diseases |
| - Non-Hodgkin's lymphoma - Multiple myeloma - Histiocytosis - Bone tumors/metastases |
| Hypereosinophilic syndrome |

**Table S13: B-Findings (Borderline-Benign) for the diagnosis of** **SSM**

| **B-Findings*** | **Clinical Manifestation** |
| --- | --- |
| 1. Large mast cell mass | At bone marrow biopsy > 30% of cells are mast cells grouped or not in aggregates, and/or serum tryptase level > 200 µg/L, and/or VAF KIT in blood >10 according to the WHO criteria 2022  At bone marrow biopsy > 30% of cells are mast cells grouped, and/or not in aggregates, and serum tryptase level > 200 µg/L according to the ICC criteria 2022 |
| 1. Bone marrow dysplasia | Signs of dysplasia of other hematopoietic lineages but without diagnostic arguments in favor of another hematologic malignancy (CBC normal) |
| 1. Organomegaly | Hepatosplenomegaly without organ abnormalities and/or lymphadenopathy; the size of lymphadenopathy is > 1cm in the ICC criteria 2022 |

*Two B-findings are necessary to enable diagnosis of borderline systemic mastocytosis (smoldering SM)

**Table S14: C-Findings (Consider Cytoreduction) for the diagnosis of aggressive SM** [60]

| **C-Findings** | **Clinical manifestation** |
| --- | --- |
| 1. Bone marrow abnormality due to mast cell infiltrate | ≥ 1 Cytopenia defined by neutrophils <1.0 × 10^9^/L, Hemoglobin <10 g/dL and/or platelets <100 × 10^9^/L |
| 1. Hepatomegaly with impaired organ function | Ascites and/or PHT |
| 1. Bone damage | Osteolytic lesion > 2 cm with or without pathological fracture |
| 1. Splenomegaly | Hypersplenism, cytopenia |
| 1. Malabsorption with weight loss and hypoalbuminemia | Gastrointestinal mast cell infiltrate |

PHT = portal hypertension

**Table S15: Examinations** **necessary prior to initiation of basic treatments for ISM patients with disability and for therapeutic monitoring**

| **Treatment** | **Pretherapeutic assessment** |
| --- | --- |
| Omalizumab | - CBC, platelets - Serum tryptase - Total IgE - Serum calcium - BMD |
| Tyrosine kinase inhibitors | - CBC, platelets - Serum tryptase - Complete liver assessment - Beta hCG - Serum calcium - ECG - BMD |
| Bisphosphonate or other treatment targeting bone | - CBC - Serum calcium - TSH - Bone alkaline phosphatase - BMD - Dental panoramic X-ray - Stomatology/odontology consultation for local treatment of infectious sites |

ALT: alanine aminotransferase; AST: aspartate aminotransferase; BMD: bone mineral density; CBC: complete blood count; Gamma GT: gamma glutamyl transpeptidase

* Complete liver assessment = AST, ALT, gamma GT, alkaline phosphatase, bilirubin

**Table S16: Precautions when starting disease-modifying treatments for mastocytosis**

| Tyrosine kinase inhibitors |
| --- |
| - Considering the risk of reactions to several tyrosine kinase inhibitors with excessive exposure to sun [96–98]; it appears prudent to recommend external sun protection to all patients undergoing this type of treatment. - Regarding midostaurin, particular attention must be paid to the risk of hepatopathy and the capsules should be opened 1 hour before administration to reduce risk of digestive adverse effects. - Regarding avapritinib, particular attention must be paid to patients with low platelet counts due to crossing of the blood‒brain barrier and the attendant risk of cerebral hemorrhage. However, this is not a risk for patients presenting forms of mastocytosis treated in the present PNDS (nonadvanced mastocytosis). - Regarding masitinib, elenestinib and bezaclastinib, we do not have sufficient information or hindsight to call attention to any specific points. |

**Table S17: Examinations needed to monitor basic treatments for SM in patients with disability**

| **Treatment** | **Therapeutic assessment** |
| --- | --- |
| Omalizumab | - CBC, platelets - Total IgE - Serum tryptase every 6 months - Serum calcium every 6 months - BMD every 24 to 36 months |
| Bisphosphonate | - CBC, platelets - Serum calcium - BMD every 24 to 36 months |

ALT: alanine aminotransferase; AST: aspartate aminotransferase; BMD: bone mineral density; CBC: complete blood count; D: day; Gamma GT: gamma glutamyl-transpeptidase

* Complete liver assessment = AST, ALT, gamma GT, alkaline phosphatase, bilirubin

**Figure S1: Classification of mast cell activation syndrome (adapted from Gülen et al.** [50]**)**


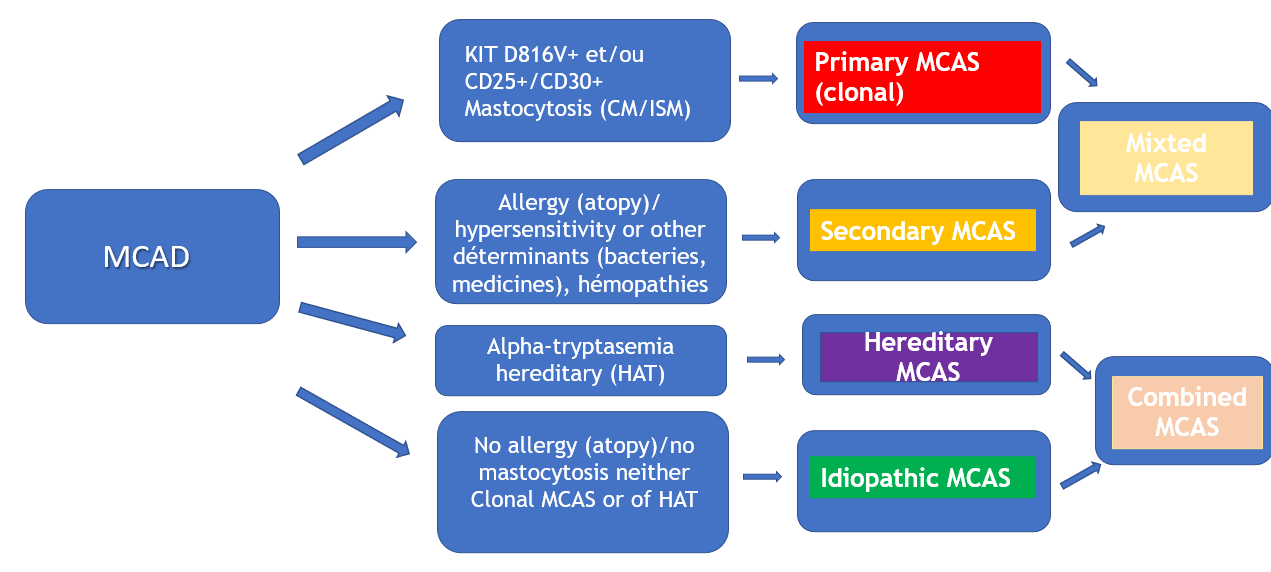

Supplement: Supplementary file 1 — Supplementary material 1 [file 13023_2025_3764_MOESM1_ESM.docx]
